# The Role of Knockout Olfactory Receptor Genes in Odor Discrimination

**DOI:** 10.3390/genes12050631

**Published:** 2021-04-23

**Authors:** Maria Pina Concas, Massimiliano Cocca, Margherita Francescatto, Thomas Battistuzzi, Beatrice Spedicati, Agnese Feresin, Anna Morgan, Paolo Gasparini, Giorgia Girotto

**Affiliations:** 1 Institute for Maternal and Child Health—IRCCS, Burlo Garofolo, 34127 Trieste, Italy; mariapina.concas@burlo.trieste.it (M.P.C.); massimiliano.cocca@burlo.trieste.it (M.C.); paolo.gasparini@burlo.trieste.it (P.G.); giorgia.girotto@burlo.trieste.it (G.G.); 2 Department of Medicine, Surgery and Health Sciences, University of Trieste, 34149 Trieste, Italy; margherita.francescatto@burlo.trieste.it (M.F.); beatrice.spedicati@burlo.trieste.it (B.S.); agnese.feresin@burlo.trieste.it (A.F.); 3 Department of Life Sciences, University of Trieste, 34127 Trieste, Italy; THOMAS.BATTISTUZZI@studenti.units.it

**Keywords:** whole-genome sequencing, odor discrimination, olfactory receptors genes, loss of function variants, human knockouts, gene expression

## Abstract

To date, little is known about the role of olfactory receptor (OR) genes on smell performance. Thanks to the availability of whole-genome sequencing data of 802 samples, we identified 41 knockout (KO) OR genes (i.e., carriers of Loss of Function variants) and evaluated their effect on odor discrimination in 218 Italian individuals through recursive partitioning analysis. Furthermore, we checked the expression of these genes in human and mouse tissues using publicly available data and the presence of organ-related diseases in human KO (HKO) individuals for OR expressed in non-olfactory tissues (Fisher test). The recursive partitioning analysis showed that age and the high number (burden) of OR-KO genes impact the worsening of odor discrimination (*p-*value < 0.05). Human expression data showed that 33/41 OR genes are expressed in the olfactory system (OS) and 27 in other tissues. Sixty putative mouse homologs of the 41 humans ORs have been identified, 58 of which are expressed in the OS and 37 in other tissues. No association between OR-KO individuals and pathologies has been detected. In conclusion, our work highlights the role of the burden of OR-KO genes in worse odor discrimination.

## 1. Introduction

Animals, including humans, perceive themselves and everything surrounding them thanks to their senses, and only the sensory coding allows species to make crucial decisions that lead to a specific behavioral response [1]. Among the sensory systems, the sense of smell is the most ancient and gives us the ability to perceive odorants, which are mixtures of different chemical molecules. This ability is present in micro-organisms as well as in complex species such as mammals. However, during evolution, human beings’ increasing reliance of other senses, such as vision, has decreased our sense of smell [2]. Nevertheless, the OS is the designated machinery for recognizing and elaborating conscious olfactory stimuli allowing humans to discriminate more than a trillion odorant stimuli [3,4]. Anatomically, the OS extends from the nose’s superior part to the brain’s higher structures. The crucial component is the olfactory epithelium (OE) which is highly specific for each species and is deeply connected to their reliance on the sense of smell. The OE is characterized by several types of cell, the most important of which are the olfactory sensory neurons (OSNs), bipolar neurons capable of regeneration [3]. The precise mechanism of OSN regeneration, maturation, and the subsequent axonal connection is still unknown, but this turnover mechanism decreases progressively over time leading to age-related olfactory function loss [5]. On each cilium, OSNs express one OR gene, which allows the interaction with different odorants [3]. The exact mechanism of odor coding is still undeciphered, but the odorants’ identification seems to work as a “combinatorial code” in which one OR can identify several odorants while different odorants are recognized by multiple combinations of receptor [6].

Olfactory receptors (ORs) belong to the superfamily of G-protein coupled receptors (GPCR). The number of OR genes and pseudogenes in the genome varies significantly between different species [7,8,9]. It does not always correlate with their smell ability, suggesting that other factors may be involved (e.g., larger surfaces of OE in dogs, a high number of glomeruli in humans, etc.) [10,11,12]. Mammals have about 1000 olfactory genes, while other living organisms such as fishes no more than 100 [9]. Humans have 851 OR genes, but only 45% of them are functional [13]. In humans, the OR genes are distributed in clusters located on all chromosomes, except for chromosomes 20 and Y [14]. Recent evidence suggests that, apart from the OE, OR are widely expressed in several other tissues such as the brain, tongue, testis and liver [15]. The OR genes are intron-less and, despite not being individually expressed in each OSN, they are expressed by a single allele [13]. An inter-individual phenotypic variation in the olfactory function within members of the same species suggests a different pattern of genetic variants in ORs and an influence of both environment and demographic factors [15,16,17].

Among non-genetic components, it is well established that aging is a driver factor involved in olfactory decay [17]. Moreover, other conditions such as neurodegenerative diseases [18], head trauma [19], brain tumors [20], brain surgery [21], and infections [22] have been proved to play a role in olfactory dysfunction. 

As for OR genetic variations, they probably contribute to the diversity of odorant-specific sensitivity phenotypes. For example, the role of two variants (rs61729907 or R88W, and rs5020278 or T133M) within the *OR7D4* gene which impair the individual ability to perceive androstenone (5a-androst-16-en-3-one) is well known [23]. Recently, Gisladottir and colleagues [24], through a whole-genome sequencing analysis discovered a common variant in *OR6C70* associated with a higher intensity and naming of licorice odor (trans-anethole). Other studies highlighted the role of OR variants on specific odorants [25,26,27,28,29,30,31]. However, there is still a lack of data regarding the hundreds of receptors’ interactions with the multitude of odorous molecules. Therefore, more efforts are needed to increase our knowledge of the genetic basis of this sense. In this light, the possibility of studying individuals defined as human knockout (HKO) (i.e., carriers of biallelic loss of function (LoF) variants) can give the unprecedented opportunity further to explore the role and the function of OR genes. 

In this study, we hypothesized that the amount of knockout OR genes (KO-OR) could impact the individual general smell ability, without focusing on a single OR or single odorant. Analyzing data from two Italian genetic isolates, we identified carriers of biallelic LoF variants in OR genes (i.e., OR-human knockout (HKO)), and investigated their relationship with odor discrimination data measured through the Sniffin’ Sticks test. The main aim was to understand better the these genes’ role in smell ability investigating the possible correlation between the burden of OR-KO genes and the smell ability. As secondary objects, we studied the expression pattern of the OR-KO genes in the OS and other tissues of both humans and mice and the possible development of organ-related diseases in individuals’ OR-KO for proteins expressed in the non-olfactory epithelium.

## 2. Results

**Figure 1:**
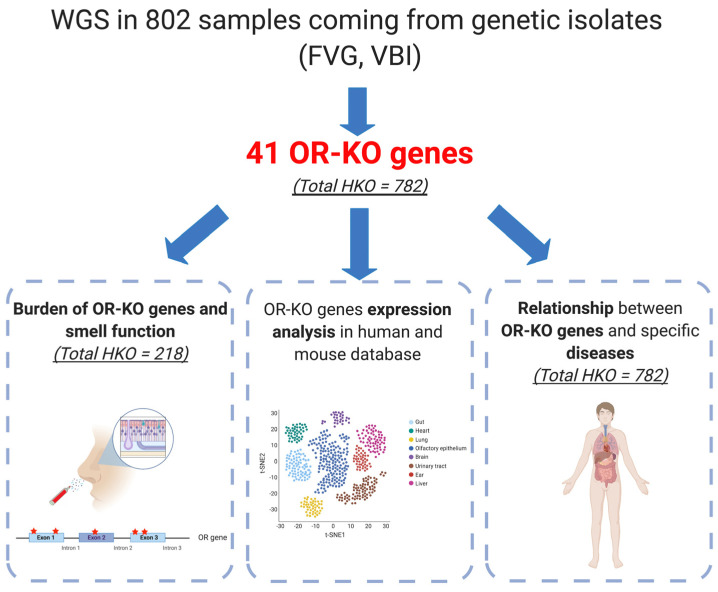
Workflow of the study. The picture summarizes the general workflow applied in the present study. Briefly, whole genome sequencing (WGS) data of 802 samples have been checked, searching for olfactory receptors (OR) genes carrying loss of function (LoF) variants. Data analysis led to the identification of 41 OR-KO (Olfactory Receptor knockout) genes in 782 subjects. Among those individuals, Sniffin’ Sticks test data were available for a total of 218 persons. The association between the burden of OR-KO genes and odor discrimination was tested, together with the analysis of OR-KO genes’ expression in human and mouse tissues and the correlation between OR-KO genes and specific diseases.

The Figure 1 shows the workflow of the study.

### 2.1. Dataset Overview and Characterization of Olfactory Receptor Knockout (OR-KO) Variants

Briefly, as reported in our previous work [32], low coverage whole genome sequencing (WGS) data from Italian individuals was analyzed with an in-house bioinformatics pipeline based on GATK (Genome Analysis Toolkit) best practices [33] to identify common and rare genetic variants. Eight hundred and two individuals belonging to two Italian geographically distinct areas (n = 378 for the Friuli Venezia-Giulia (FVG), n = 424 for Val Borbera (VBI) cohorts) have been selected and investigated for homozygous LoF variants involving ORs. This research resulted in a list of 42 LoF variants in 41 OR genes and a total of 782 HKO (372 in FVG and 410 in VBI—defined as individuals carrying at least one homozygous LoF variant). Among these 42 variants, 14 (33.3%) were classified as stop gain and 28 (66.6%) as frameshift. The frequency of the alternative allele ranged from 0.004 (rs564566592) to 0.77 (rs10838851), and two LoFs were not present in the FVG cohort (11_5080307_AT_A and rs147062602). The comparison with data from the 1000 Genomes Project phase3 [34] and gnomAD v.2.1.1 [35] showed that the allele frequency distribution of the variants we selected was consistent with the general European population’s allele frequency spectrum. The identified variants’ complete characteristics were detailed in Table 1 and Supplementary Tables S1–S3. By comparison with the hORMdb database [36], we found information on 39 variants belonging to genes comprising 13 out of 18 OR families. All but one (14_20666175_C_CA/rs55781225) were annotated as affecting all gnomAD populations. Out of 39 putative LoF variants, seven are annotated as affecting the functional core, and two as affecting the corresponding OR’s binding cavity (as defined in [35]). To 16 variants, a negative amino acid substitution score was assigned (two of them also affected the binding cavity and one the functional core). Therefore, we concluded that at least 22 variants could impact on the binding of odorant molecules or the receptor structural integrity (Table S4).

### 2.2. Relationship between OR-KO Genes’ Burden and Smell Performance

After applying the exclusion criteria detailed in the Methods section (e.g., previous neurodegenerative disease diagnosis), 218 subjects with Sniffin’ Sticks test (93 belong to VBI and 125 to FVG cohorts) were included in the study. Their features are summarized in Table 2.

**Figure 2:**
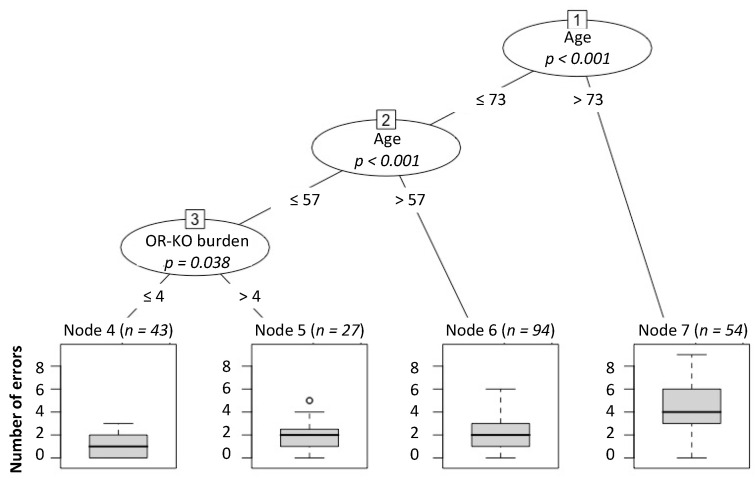
Binary tree computed by conditional recursive partitioning of the effect of OR-KO’s burden, sex, age, and population on the smell performance measured as mistakes in Sniffin’ sticks test. Smell errors were influenced by the combination of age and OR-KO genes’ burden. This analysis splits the sample into four final groups (labelled as Nodes 4–7). The group labelled Node 7 (n = 54), consisting of individuals aged >73, showed a median number of errors of 4 (IQR: 3–6). The group labelled Node 6 (n = 94), identified by individuals aged between 57 and 73, showed a median number of errors of 2 (IQR: 1–3) while for individuals aged ≤ 57 belonging to the Node 5 (OR-KO burden > 4, n = 27) and Node 4 (OR-KO burden ≤ 4, n = 43), the median number of errors was 2 (IQR: 1–2.5) and 1 (IQR: 0–2) respectively. *p* = *p*-value.

The hypothesis that an increasing number of OR-KO carried by an individual could impact the sense of smell (evaluated as the number of mistakes made in the odor discrimination test) was investigated using conditional inference tree analysis. As reported in Figure 2, this analysis showed that age and the OR-KO burden significantly influenced the number of errors, while the model was not influenced by sex or population (adjusted *p*-value > 0.05). In particular, the first variable affecting smell was age (node 1: 73 years cutoff, *p*-value < 0.001; node 2: 57 years cutoff, *p*-value < 0.001), while the second one was the OR-KO genes burden (node 3: cutoff 4 OR-KO, *p*-value 0.038). This partition led to four final subgroups (indicated as the terminal nodes labeled 4, 5, 6 and 7 in Figure 2), clearly proving that, from node 4 to node 7, there was an increasing number of errors due to both the high burden of OR-KO and aging.

### 2.3. Expression Patterns of OR-KO Genes

To investigate OR-KO genes’ expression, we used publicly available data on human and mouse expression in multiple cell lines and tissues. The results are reported in Table 3.

Human RNA-seq data extracted from Saraiva et al. [37] revealed that 33 out of 41 OR genes (80.5%) had detectable expression in human olfactory tissue, with expression spanning from 0.35 to 160.36 normalized counts (NCs). In particular, 28 showed evidence of robust expression (>1 NCs). Moreover, according to the Human Protein Atlas (HPA) database, 27 genes (65.9%) were expressed, at least, in another tissue. 

From the list of 41 human ORs, we identified 60 putative mouse homologs through the Mouse Genome Informatics (MGI) resource. OS expression data [32] showed robust expression (>1 NCs) for 51 out of the 60 identified mouse homologs (85%). The MGI database confirmed expression in the OS for 58 of 60 mouse homologs, with 37 of them (63.8%) being expressed in tissues other than OE.

### 2.4. Relationship with Pathologies

Given the expression of the investigated genes in tissues other than OS, the presence of pathologies in HKO individuals was investigated. We focused on the FVG cohort analysis since this was the subset of individuals with the most curated pathology data available. The analysis did not identify, after Bonferroni correction for multiple testing, pathologies significantly more frequent in HKO subjects than the remainder of the population.

## 3. Discussion

Although the olfactory sense’s molecular bases are relatively well understood, there is still a considerable lack of knowledge of the contribution of the specific genes involved. Therefore, it is vital to explore further this sense considering that smell ability deficits are crucial/critical signs for the early diagnosis of neurodegenerative disorders [18,38,39]. Several works have already highlighted the effect of variants in OR genes on the perception of smell [23,24,25,26,27,28,29,30,31], but, to our knowledge, no studies evaluate the effect of the burden of OR-KO genes on smell ability. 

In this light, we combined WGS data of a large cohort of samples with detailed phenotypic data to unravel this unsolved issue. In particular, thanks to the availability of WGS of 802 Italian samples, we identified 41 OR-KO genes (i.e., genes for which we identified individuals carrying LoF variants in the homozygous state). We evaluated their effect on the smell capacity in 218 individuals, for whom the odor discrimination evaluation was assessed through the Sniffin’ Sticks test. For the first time, we demonstrated that OR-KO genes’ burden was significantly associated with a worse smell performance in young subjects (i.e., aged ≤57 years). More precisely, the younger individuals carrying more than 4 OR-KO genes showed a worse performance in the odor identification test. Interestingly, although the OR-KO genes are 41, 4 is the median number of OR-KO genes per individual. This result might be related to these mutational events cumulative effects (that simultaneously turn off the expression of a series of OR genes), as also hypothesized for other conditions [40,41]. Moreover, the data made available by the recently published work of Jimenez et al. [35] allowed us to conclude that at least 22 HKO variants could impact the binding of odorant molecules or the receptor structural integrity. This last information suggested that an approach based on the burden test can help determine whether multiple homozygous LoF variants influence the ability to recognize the odors. Our data agreed with previous ones showing that age was a major player in the progressive worsening of the sense of smell, overcoming the genetic factors in older individuals (i.e., aged >57 years) [25]. 

Regarding the OR-KO expression patterns, it has been highlighted that many OR genes are expressed in several structures other than the OS in both humans and mice, thus suggesting that they may exert a role in non-chemosensory tissues. We looked for any relationship between OR-KO genes and specific pathologies, but we did not find any disorders significantly more frequent in OR-KO subjects than in the rest of the population. Several possible explanations could justify this lack of association, including the small number of cases and the lack or incompleteness of data on tissue-specific OR gene expression in public databases. Information about tissue-specific expression was not feasible for many ORs, and therefore, in this case, it was not possible to speculate on any association with a particular disease. On the other hand, regarding ORs whose pattern of expression was publicly available, it could be argued that data were still widely incomplete. Most ORs were apparently over-expressed in the male or female reproductive system, in bone-marrow-derived cells, and the brain, with a relative absence of expression in all other tissues.

In general, our study, for the first time, reported WGS data combined with the smell phenotype of a selected cohort of Italian genetic isolates. Our results allowed us to identify an interesting association between OR-KO genes’ burden and less smell performance in younger people, suggesting the importance of the genetic background in determining human olfactory capability. Present data also corroborated the hypothesis that aging processes are more relevant than the individual genetic background in impairing smell ability. Further studies on larger datasets are needed, including other population cohorts, although data from individuals with WGS and information on the sense of smell are relatively limited.

## 4. Materials and Methods 

### 4.1. Identification of OR-KO Genes and Comparison with External Databases

A subset of HKO variants involving OR genes were selected from the data generated in [30] for further analysis. HKO variants were defined as LoF variants presenting with a CADD (Combined Annotation Dependent Depletion) score ≥ 20 at homozygous state in at least one individual of at least one population. We defined “burden of OR-KO genes” as the total number of OR genes KO per individual and compared alternative allele (ALT) frequencies of HKO variants with data from 1000 Genomes Project phase 3 [34] and gnomAD v.2.1.1 [35] using the R implementation of the Chi-squared test. We extracted information about topological annotations from the Human Olfactory Receptor Mutation Database (hORMdb) [36].

### 4.2. Clinical Evaluation

The clinical evaluation of all subjects enrolled in the study was characterized by evaluating hundreds of functional parameters, including clinical, biochemical data, and bone densitometry. We performed a sensory evaluation focused on the analysis of senses (hearing, taste, smell, and vision—for details on the smell functionality assessment, see next section), a cardiovascular, neurological, orthodontic evaluation, a detailed personal and familial history with more than 200 questions asked to each subject. All parameters were systematically collected by professional and trained staff according to standardized protocols; participants were also required to fill in a questionnaire on health-related topics, including diet, lifestyles, and physical activity.

### 4.3. Smell Functionality Assessment

Smell functionality of each subject was assessed through the “Sniffin’ Sticks test” (Screening 12 test, Burghardt Messtechnik GmbH, Wedel, Germany), a smell discrimination test which contains 12 “Sniffin’ sticks”, felt-tip pens with precise odorants to be recognized [42]. The test is based on the discrimination of every-day odors (i.e., peppermint, fish, coffee, banana, orange, rose, lemon, pineapple, cinnamon, cloves, leather and licorice) through a “multiple-forced-choice” method. Individuals with incomplete data about sex, age, and answers to all 12 sticks were excluded from the analyses. Furthermore, individuals with conditions that could affect smell performance, such as respiratory (asthma, sinusitis, septal surgery, etc.) or neurological diseases [43,44], were ruled out. 

### 4.4. Relationship between Smell Performance and the Burden of OR-KO Genes

Conditional inference trees analysis (R “party” package) was used to test the influence of the burden of OR-KO genes (in addition to age, sex, and population) on smell functionality (number of errors in Sniffin’ Sticks test) [45,46]. This statistical method is efficacious in studies in which there are subgroups with different levels of response to the variables explained. Briefly, the following algorithm was applied [47]: (1) to test the global null hypothesis of independence between any of the explanatory variables and the response. It was interrupted if this hypothesis could not be rejected based on a Bonferroni correction (α = 0.05). Otherwise, it selected the explanatory variable with the strongest association to the response; (2) implementing a binary split in the selected explanatory variable; (3) recursively repeating steps (1) and (2). 

### 4.5. Expression of ORs in Human and Mouse

Human and mouse normalized expression data were downloaded from the supplementary materials of the mammalian olfactory mucosae transcriptomic atlas [37]. The data included normalized expression averages across three human and three mouse OE samples. The Human Protein Atlas (HPA) [48] was interrogated to verify the evidence of OR genes expression in non-OE tissues and the genes with expression below 1 normalized count were considered not expressed. The Mouse Genome Informatics (MGI) resource [49] was used to identify mouse homologs/orthologues and assess expression patterns of the homologs detected in the OS and other tissues.

### 4.6. Relationship with Pathologies

We asked if there was a significantly over-represented pathology in individuals carrying the KO genes than the rest of the sequenced population. The analysis focused on the sequenced individuals from the FVG cohort for whom detailed and curated anamnestic information was available (pathologies classified according to the International Classification ICD-10). For each OR gene, we extracted the pathologies observed in the group of KO individuals. For each disease/phenotype, a case-control study was carried out comparing its recurrence in HKO cases versus the group of individuals non-HKO (R implementation of the Fisher exact test, significance threshold set at Bonferroni corrected *p*-value < 0.001).

## Figures and Tables

**Table 1:** Characteristics of the homozygous LoF variants in OR genes identified in our Italian cohorts. All data are aligned to the human genome reference build 37 (GRCh37), and VEP (Variant Effect Predictor, https://www.ensembl.org/info/docs/tools/vep/index.html) version 90 was used to determine the variant consequence. *Chr* = chromosome, *Pos* = position, *Ref* = reference allele, *Alt* = alternative allele, *Freq* = frequency of the reference allele, *KO* = knockout, *N* = number, *FVG* = Friuli-Venezia Giulia, *VBI* = Val Borbera. The last two columns refer to the number of KO individuals in FVG and VBI (“*KO FVG/VBI*”) and the number of human knockout (HKO) with information of Sniffin’ Sticks test and used in regression tree analysis (“*N smell FVG/VBI*”).

Gene	Isoform	cDNA Change	Protein Change	Chr	Pos	Ref	Alt	rsID	Freq FVG/VBI	KO FVG/VBI	N Smell FVG/VBI
*OR10J1*	NM_012351.3	c.759T > A	p.(Cys253*)	1	159410340	T	A	rs12409540	0.0675/0.1144	1/6	0/1
*OR2W3*	NM_001001957.2	c.893dup	p.(Ala300Glyfs*?)	1	248059779	G	GA	rs80255919	0.1614/0.0873	13/2	1/0
*OR2T4*	NM_001004696.1	c.757del	p.(Ile253Serfs*8)	1	248525638	CA	C	rs34079073	0.5106/0.5224	110/120	40/20
*OR5K4*	NM_001005517.1	c.901del	p.(Ile301Leufs*2)	3	98073591	TA	T	rs11288615	0.6177/0.5224	141/121	57/31
*OR5K3*	NM_001005516.1	c.904dup	p.(Ile302Asnfs*?)	3	98110406	G	GA	rs79045298	0.6098/0.5377	138/129	55/33
*OR5K2*	NM_001004737.1	c.654T > A	p.(Tyr218*)	3	98217178	T	A	rs55639376	0.1204/0.1392	3/11	2/1
*OR2V2*	NM_206880.1	c.320_323del	p.(Cys107Leufs*30)	5	180582256	TTGTC	T	rs140598308	0.0582/0.1014	2/3	0/0
*OR13C5*	NM_001004482.1	c.926del	p.(His309Profs*3)	9	107360768	GT	G	rs11314210	0.1706/0.1321	13/3	5/1
*OR1J1*	NM_001004451.1	c.705C > A	p.(Cys235*)	9	125239501	G	T	rs45579335	0.0066/0.0224	0/1	0/0
*OR1J2*	NM_054107.1	c.312dup	p.(Ile105Tyrfs*5)	9	125273385	A	AT	rs145911830	0.1204/0.1439	3/8	2/2
*OR13A1*	NM_001004297.3	c.805dup	p.(Tyr269Leufs*66)	10	45799065	T	TA	rs35302355	0.0608/0.0377	5/1	3/1
*OR51T1*	NM_001004759.2	c.551_552insCACCACCC	p.(Glu185Thrfs*5)	11	4903673	T	TACCACCCC	rs564566592	0.004/0.013	0/1	0/0
*OR52J3*	NM_001001916.2	c.907C > T	p.(Arg303*)	11	5068662	C	T	rs57026471	0.1098/0.1285	5/7	0/1
*OR52E2*	NM_001005164.2	c.551del	p.(Met184Argfs*25)	11	5080307	AT	A	-	(null)/0.0106	(null)/1	0/0
*OR52A1*	NM_012375.2	c.804dup	p.(Ser269Valfs*13)	11	5172795	A	AC	rs112098990	0.2447/0.3113	27/44	6/17
*OR51B5*	NM_001005567.3	c.197_213del	p.(Ala66Glyfs*48)	11	5364541	CCAGCCCCAGGTCTGTGG	C	rs147062602	(null)/0.0377	(null)/1	0/0
*OR51J1*	NM_001348224.1	c.567_570dup	p.(Cys191Ilefs*8)	11	5424387	T	TTATC	rs113047337	0.1005/0.092	7/6	1/3
*OR51Q1*	NM_001004757.2	c.706C > T	p.(Arg236*)	11	5444136	C	T	rs2647574	0.3532/0.4021	55/79	20/19
*OR51I1*	NM_001005288.2	c.43C > T	p.(Gln15*)	11	5462702	G	A	rs16930998	0.0172/0.0212	1/1	1/0
*OR51I2*	NM_001004754.2	c.714_715dup	p.(Asn239Thrfs*18)	11	5475431	T	TCA	rs35301588	0.3704/0.309	54/46	15/12
*OR52D1*	NM_001005163.2	c.605_608dup	p.(Thr204Alafs*33)	11	5510540	G	GGGCT	rs576495879	0.1442/0.1568	17/11	5/5
*OR52N4*	NM_001005175.3	c.514A > T	p.(Arg172*)	11	5776484	A	T	rs4910844	0.2341/0.3208	23/42	8/9
*OR4X1*	NM_001004726.1	c.819T > A	p.(Tyr273*)	11	48286231	T	A	rs10838851	0.7659/0.6344	223/165	76/38
*OR4C11*	NM_001004700.2	c.469C > T	p.(Gln157*)	11	55371381	G	A	rs75423534	0.0754/0.0955	14/18	7/5
*OR4P4*	NM_001004124.2	c.189C > G	p.(Tyr63*)	11	55406022	C	G	rs76160133	0.1296/0.1922	21/45	7/4
*OR8I2*	NM_001003750.1	c.867C > G	p.(Tyr289*)	11	55861650	C	G	rs61887097	0.1124/0.0849	7/5	3/1
*OR5M11*	NM_001005245.1	c.378T > A	p.(Tyr126*)	11	56310356	A	T	rs17547284	0.119/0.0896	10/4	4/1
*OR5M10*	NM_001004741.1	c.347_354del	p.(Ala116Glyfs*37)	11	56344843	CCATTGAAG	C	rs148438199	0.119/0.0873	10/4	4/1
*OR5M1*	NM_001004740.1	c.429_432del	p.(Cys143Trpfs*19)	11	56380546	CCAGA	C	rs71931749	0.2302/0.263	21/24	13/7
*OR6Q1*	NM_001005186.2	c.685del	p.(Leu229Cysfs*21)	11	57799108	AC	A	rs34846253	0.2487/0.2229	25/20	8/5
*OR10D3*	NM_001355213.1	c.756T > G	p.(Tyr252*)	11	124056732	T	G	rs2512227	0.4987/0.5696	90/135	27/33
*OR8B3*	NM_001005467.1	c.550dup	p.(Leu184Profs*23)	11	124266697	A	AG	rs201661436	0.0635/0.0649	0/3	0/2
*OR10AD1*	NM_001004134.1	c.199_200insG	p.(Leu67Argfs*56)	12	48596875	C	CA	rs79650217	0.2063/0.2406	19/28	6/8
*OR9K2*	NM_001005243.1	c.38del	p.(Leu13Cysfs*22)	12	55523586	AT	A	rs58036029	0.3823/0.3278	59/43	21/13
*OR6C74*	NM_001005490.1	c.184C > T	p.(Arg62*)	12	55641255	C	T	rs4522268	0.377/0.3243	56/42	21/13
*OR6C1*	NM_001005182.1	c.24dup	p.(Glu9Argfs*10)	12	55714406	C	CA	rs5798345	0.4405/0.3892	79/64	26/10
*OR6C76*	NM_001005183.1	c.933del	p.(Lys311Asnfs*?)	12	55820958	CA	C	rs57387180	0.1772/0.2205	12/26	6/6
*OR4L1*	NM_001004717.1	c.248_266del	p.(Ile83Thrfs*10)	14	20528448	TCATAGATTTGCTCACTGAC	T	rs33965693	0.3981/0.3561	64/54	25/13
*OR11G2*	NM_001005503.1	c.687_688dup	p.(Gly230Lysfs*4)	14	20666175	C	CA	rs55781225	0.668/0.6568	170/183	71/32
*OR2C1*	NM_012368.3	c.818del	p.(Phe273Serfs*13)	16	3406756	GT	G	rs142397376	0.0886/0.0837	1/1	0/0
*OR7G3*	NM_001001958.1	c.928_929insACTAT	p.(Ser310Tyrfs*?)	19	9236698	G	GATGGT	rs111867493	0.2791/0.2925	27/40	10/11
*OR7G3*	NM_001001958.1	c.710del	p.(Ala237Valfs*9)	19	9236916	AG	A	rs75266995	0.0304/0.0519	0/3	0/0

**Table 2:** Characteristic of individuals included in regression tree analysis. The table provides details of individual characteristics of subjects included in the regression tree analysis, indicating sex, age, number of errors in Sniffin’ Sticks test, and the classification of individuals in normosomic, hyposomic, anosmic, the numbers of individuals for each OR-KO gene and the number of OR-KO genes.

N (males %)	218 (43.6%)
Age (y), mean (SD)	61.9 (15.3)
Number of errors in Sniffin’ Sticks test, median (IQR)	2.0 (1.0–4.0)
Normosmic *, %	34.9
Hyposmic, %	50.9
Anosmic, %	14.2
Number of individuals for each OR-KO gene **, [range], median (IQR)	[0–114], 8.0 (1.0–28.75)
Number of OR-KO genes per individual, [range], median (IQR)	[0–11], 4.0 (3.0–5.0)

* Individuals are classified normosmic if the number of errors was <2, hyposmic if the number of errors was between 2 and 4 and anosmic if the number of errors was >4. ** The number of individuals carrying each specific OR-KO gene is indicated in Table 1. y = years; IQR = interquartile range.

**Table 3:** Expression patterns of the OR-KO genes in different human and mouse tissues. The genes with robust expression (>1 NCs, NC = normalized counts) in human OE are indicated in bold. Expression human OE: average expression across three human OE samples from [37] measured as NC. Expression human tissues: list of tissues with expression above 1 NX (NX = Normalized eXpression) reported in the HPA (Human Protein Atlas); 0: no tissue with expression above 1NX; NA: gene not found in database. Mouse Gene Symbol: most likely mouse homolog identified through the MGI (Mouse Genome Informatics) database; note that each human OR gene can be associated to one, multiple, or no homolog (in this case NA). Expression Mouse OE: average expression across three mouse OE samples from [37] measured as NC. Expressed Mouse OS indicates whether MGI reports expression in a tissue of the OS. Expression Mouse Tissues: indicates that non-OS tissues expression is reported in MGI; NA non expression in non-OS tissues.

Human Gene Symbol	Expression Human OE (Saraiva et al., 2019)	Expression Human Tissues (Human Protein Atlas)	Mouse Gene Symbol (Mouse Genome Informatics)	Expression Mouse OE (Saraiva et al., 2019)	Expressed Mouse OS (Mouse Genome Informatics)	Expression Mouse Tissues (Mouse Genome Informatics)
***OR10J1***	4.37	Testis, granulocytes	Olfr418 (1)	NA	Yes	Alimentary system
***OR2W3***	4.7	Bone marrow, thyroid gland, cerebral cortex, hypothalamus, basal ganglia	Olfr322	NA	Yes	NA
			Olfr317	24.29	Yes	Nervous system, reproductive system
***OR2T4***	2.22	Prostate, cervix uterine, cerebral cortex	Olfr331	0.28	No	NA
			Olfr224	303.89	Yes	Hemolymphoid system, reproductive system
			Olfr325	245.05	Yes	Embryo ectoderm, auditory system, reproductive system
			Olfr328	287.48	Yes	Reproductive system
			Olfr329 (2)	395.85	Yes	Early conceptus, endocrine system, hemolymphoid system, reproductive system
			Olfr330	341.8	Yes	Alimentary system, auditory system, endocrine system, reproductive system
*OR5K4*	0	0	Olfr180	188.79	Yes	Reproductive system
*OR5K3*	0	0	Olfr175 (3)	NA	Yes	Liver and biliary system
			Olfr195	506.03	Yes	Urinary system
***OR5K2***	7.52	Skeletal muscle, cerebellum, skin, lung, colon	Olfr177	142.22	Yes	NA
***OR2V2***	38.24	Granulocytes, bone marrow, fallopian tube	Olfr1396	203.03	Yes	Cardiovascular system, connective tissue, hemolymphoid system, integumental system, limbs, liver and biliary system, musculoskeletal system, urinary system
***OR13C5***	2.07	0	Olfr452	78.19	Yes	Auditory system
***OR1J1***	2.9	Salivary gland, testis, bone marrow, granulocytes	Olfr3	34.88	Yes	Auditory system, reproductive system
***OR1J2***	4.89	Urinary bladder, epididymis, testis	Olfr348	22.23	Yes	NA
***OR13A1***	43.39	Urinary bladder, spleen, lymph node, tonsil, B-cells	Olfr211	404.96	Yes	NA
***OR51T1***	1.04	Prostate	Olfr574	0	Yes	Endocrine system, nervous system
***OR52J3***	1.66	0	Olfr592	36.36	Yes	Auditory system
*OR52E2*	0.71	Testis	Olfr589	1.33	Yes	Auditory system
			Olfr594	63.59	Yes	Nervous system
***OR52A1***	50.01	Granulocytes, testis, B-cells, skeletal muscle, cerebellum	Olfr68	18.3	Yes	Liver and biliary system
***OR51B5***	1.71	Epididymis, T-cells	NA	NA	NA	NA
*OR51J1*	0.71	NA	NA	NA	NA	NA
*OR51Q1*	0.94	Epididymis, cerebellum	Olfr635	36.72	Yes	NA
			Olfr638	11.5	Yes	NA
***OR51I1***	3.73	Epididymis, testis	Olfr639	46.82	Yes	Reproductive system
			Olfr640	136.96	Yes	NA
*OR51I2*	0	Granulocytes	Olfr641	15.13	Yes	Branchial arches, nervous system
***OR52D1***	1.04	Testis	Olfr646	25	Yes	NA
			Olfr691	79.3	Yes	Auditory system, nervous system, reproductive system
***OR52N4***	11.26	Spleen, small intestine, ovary, epididymis, T-Cells	Olfr503	0.66	NA	NA
			Olfr658	14.75	Yes	Nervous system, visual system
*OR4X1*	0	0	NA	NA	NA	NA
***OR4C11***	3.39	0	Olfr1201	27.04	Yes	NA
			Olfr1205	158.61	Yes	NA
			Olfr1206	206.79	Yes	NA
***OR4P4***	8.26	Bone marrow, granulocytes, skin, natural killer (NK) cells	NA	NA	NA	NA
*OR8I2*	0	0	Olfr1104	81.01	Yes	Early conceptus, reproductive system
*OR5M11*	0	Urinary bladder, testis	Olfr1028	23.72	Yes	NA
			Olfr1029	18.05	Yes	Liver and biliary system, nervous system, reproductive system
***OR5M10***	17.86	Salivary gland	Olfr1022	2.42	Yes	Nervous system
			Olfr1023	12.88	Yes	Nervous system
***OR5M1***	16.73	0	Olfr1023	12.88	Yes	Nervous system
*OR6Q1*	0	0	NA	NA	NA	NA
***OR10D3***	7.09	Testis	Olfr958	42.05	Yes	Branchial arches, nervous system
***OR8B3***	22.86	Testis	Olfr147	52.98	Yes	Early conceptus
***OR10AD1***	2.28	Pituitary gland, adrenal gland, testis, cerebellum, appendix	Olfr286	NA	Yes	Embryo ectoderm, hemolymphoid system, nervous system, reproductive system
			Olfr287	NA	Yes	Early conceptus, hemolymphoid system, nervous system, reproductive system
			Olfr288	80.43	Yes	Early conceptus, alimentary system, musculoskeletal system, reproductive system, urinary system
***OR9K2***	9.33	0	Olfr825	41.63	Yes	NA
			Olfr826	32.78	Yes	NA
*OR6C74*	0.35	0	Olfr821	95.42	Yes	NA
***OR6C1***	21.11	0	Olfr786	119.92	Yes	NA
			Olfr802	30.95	Yes	NA
***OR6C76***	5.05	Epididymis, fallopian tube	Olfr792	23.76	Yes	Nervous system, reproductive system
			Olfr798	60.66	Yes	Embryo ectoderm
			Olfr809	41.56	Yes	NA
			Olfr813	56.92	Yes	Auditory system
*OR4L1*	0.88	0	Olfr723	91.15	Yes	NA
			Olfr724	33.25	Yes	NA
***OR11G2***	160.36	Bone marrow	Olfr744	39.01	Yes	Hemolymphoid system
***OR2C1***	1.65	Fallopian tube, T-cells, spinal cord, parathyroid gland, B-cells	Olfr15	785.44	Yes	Auditory system
*OR7G3*	0	Fallopian tube	Olfr832	9.79	Yes	Reproductive system
			Olfr834	0	Yes	NA

## Data Availability

The genetic data described in this manuscript have been submitted to the European Variation Archive (EVA) in 2019 and are accessible in Variant Call Format (VCF) at the following link: https://www.ebi.ac.uk/ena/data/view/PRJEB33648 accessed on 5 June 2019).

## Supplementary Materials

Supplementary materials can be found at https://www.mdpi.com/article/10.3390/genes12050631/s1. Table S1: 1000 Genomes project alleles frequencies for each LoF variant considered in this study. Table S2: gnomAD dataset alleles frequencies for each LoF variant considered in this study. Table S3: Comparison of the allele frequency of each LoF in our populations (FVG and VBI) to the corresponding allele frequency reported in both 1000 Genomes and gnomAD populations through a Chi-squared test. Table S4: Information retrieved from hORMdb to assess the likelihood of a functional impact on the corresponding OR.
