# Supplementary material for: The Role of Knockout Olfactory Receptor Genes in Odor Discrimination
Source: Genes (Basel). 2021 Apr 23;12(5):631. doi: 10.3390/genes12050631 (PMC8145969; doi:10.3390/genes12050631)
Supplement: Supplementary file 1 [file genes-12-00631-s001.zip › genes-1170168-supplementary.pdf]

Table S1. 1000 Genome project alleles frequencies for each LoF variant considered in this study. Chr = chromosome, Pos = genomic coordinate, Ref = reference allele, Alt = alternative allele, rsID = reference SNP cluster id, AF_* = allele frequency, AC_* = allele count; AN_* = allele number. A dot indicates that the variant was not found in 1000 Genomes.

Gene	Chr	Pos	Ref	Alt	rsID	AF_AFR
OR10J1	1	159410340	T	A	rs12409540	0.0265
OR2W3	1	248059779	G	GA	rs80255919	0.1339
OR2T4	1	248525638	CA	C	rs34079073	0.3389
OR5K4	3	98073591	TA	T	rs11288615	0.2352
OR5K3	3	98110406	G	GA	rs79045298	0.208
OR5K2	3	98217178	T	A	rs55639376	0.1422
OR2V2	5	180582256	TTGTC	T	rs140598308	0.1505
OR13C5	9	107360768	GT	G	rs11314210	0.6475
OR1J1	9	125239501	G	T	rs45579335	0
OR1J2	9	125273385	A	AT	rs145911830	0.0681
OR13A1	10	45799065	T	TA	rs35302355	0.0484
OR51T1	11	4903673	T	TACCACCC	rs564566592	0
OR52J3	11	5068662	C	T	rs57026471	0.0673
OR52E2	11	5080307	AT	A	.	.
OR52A1	11	5172795	A	AC	rs112098990	0.1399
OR51B5	11	5364541	CAGCCCCAGGTCTGTG	C	rs147062602	0.1377
OR51J1	11	5424387	T	TTATC	rs113047337	0.2958
OR51Q1	11	5444136	C	T	rs2647574	0.2799
OR51I1	11	5462702	G	A	rs16930998	0.0121
OR51I2	11	5475431	T	TCA	rs35301588	0.0416
OR52D1	11	5510540	G	GGGCT	rs576495879	0.0212
OR52N4	11	5776484	A	T	rs4910844	0.1498
OR4X1	11	48286231	T	A	rs10838851	0.5461
OR4C11	11	55371381	G	A	rs75423534	0.003
OR4P4	11	55406022	C	G	rs76160133	0.0968
OR8I2	11	55861650	C	G	rs61887097	0.0121
OR5M11	11	56310356	A	T	rs17547284	0.0144
OR5M10	11	56344843	CCATTGAAG	C	rs148438199	0.003

OR5M1	11	56380546	CCAGA	C	rs71931749	0.031
OR6Q1	11	57799108	AC	A	rs34846253	0.1225
OR10D3	11	124056732	T	G	rs2512227	0.4198
OR8B3	11	124266697	A	AG	rs201661436	0.0008
OR10AD1	12	48596875	C	CA	rs79650217	0.2716
OR9K2	12	55523586	AT	A	rs58036029	0.4085
OR6C74	12	55641255	C	T	rs4522268	0.1483
OR6C1	12	55714406	C	CA	rs5798345	0.1634
OR6C76	12	55820958	CA	C	rs57387180	.
OR4L1	14	20528448	CATAGATTTGCTCACTGA	T	rs33965693	0.4849
OR11G2	14	20666175	C	CA	rs55781225	.
OR2C1	16	3406756	GT	G	rs142397376	0.003
OR7G3	19	9236698	G	GATGGT	rs111867493	0.711
OR7G3	19	9236916	AG	A	rs75266995	0.0923

AC_AFR	AN_AFR	AF_AMR	AC_AMR	AN_AMR	AF_EAS	AC_EAS	AN_EAS	AF_EUR
35	1322	0.1571	109	694	0.0278	28	1008	0.1322
177	1322	0.1931	134	694	0.0317	32	1008	0.1312
448	1322	0.4452	309	694	0.3393	342	1008	0.4811
311	1322	0.3588	249	694	0.1319	133	1008	0.5875
275	1322	0.3689	256	694	0.127	128	1008	0.5964
188	1322	0.1412	98	694	0.0526	53	1008	0.16
199	1322	0.0476	33	694	0.0159	16	1008	0.0746
856	1322	0.2277	158	694	0.5754	580	1008	0.1789
0	1322	0.0043	3	694	0	0	1008	0.0239
90	1322	0.0591	41	694	0.002	2	1008	0.0974
64	1322	0.0231	16	694	0.001	1	1008	0.0358
0	1322	0.0058	4	694	0	0	1008	0.007
89	1322	0.2853	198	694	0.0863	87	1008	0.1372
.	.	.	.	.	.	.	.	.
185	1322	0.2738	190	694	0.0278	28	1008	0.2445
182	1322	0.0274	19	694	0	0	1008	0.0457
391	1322	0.1138	79	694	0.1478	149	1008	0.1113
370	1322	0.4006	278	694	0.7024	708	1008	0.3986
16	1322	0.1023	71	694	0.3581	361	1008	0.0318
55	1322	0.2983	207	694	0.0417	42	1008	0.3708
28	1322	0.1715	119	694	0.0754	76	1008	0.1779
198	1322	0.2262	157	694	0.1974	199	1008	0.2783
722	1322	0.7406	514	694	0.5476	552	1008	0.7445
4	1322	0.1225	85	694	0.005	5	1008	0.1322
128	1322	0.2968	206	694	0.4563	460	1008	0.165
16	1322	0.0447	31	694	0.001	1	1008	0.0944
19	1322	0.0504	35	694	0.001	1	1008	0.0974
4	1322	0.049	34	694	0.001	1	1008	0.0974

41	1322	0.098	68	694	0.1617	163	1008	0.2008
162	1322	0.1153	80	694	0.0218	22	1008	0.2048
555	1322	0.4755	330	694	0.5734	578	1008	0.5318
1	1322	0.0288	20	694	0.0079	8	1008	0.0477
359	1322	0.1326	92	694	0.128	129	1008	0.2346
540	1322	0.2421	168	694	0.1141	115	1008	0.334
196	1322	0.2334	162	694	0.1171	118	1008	0.333
216	1322	0.4496	312	694	0.3413	344	1008	0.4264
.	.	.	.	.	.	.	.	.
641	1322	0.5562	386	694	0.5734	578	1008	0.3837
.	.	.	.	.	.	.	.	.
4	1322	0.0403	28	694	0.0179	18	1008	0.0716
940	1322	0.245	170	694	0.3651	368	1008	0.3141
122	1322	0.0576	40	694	0.0317	32	1008	0.0427

AC_EUR	AN_EUR	AF_SAS	AC_SAS	AN_SAS
133	1006	0.1207	118	978
132	1006	0.2025	198	978
484	1006	0.4366	427	978
591	1006	0.408	399	978
600	1006	0.3978	389	978
161	1006	0.1227	120	978
75	1006	0.0654	64	978
180	1006	0.4192	410	978
24	1006	0.001	1	978
98	1006	0.0429	42	978
36	1006	0.0082	8	978
7	1006	0	0	978
138	1006	0.0562	55	978
.	.	.	.	.
246	1006	0.3384	331	978
46	1006	0.0051	5	978
112	1006	0.1227	120	978
401	1006	0.5276	516	978
32	1006	0.1667	163	978
373	1006	0.18	176	978
179	1006	0.0706	69	978
280	1006	0.2454	240	978
749	1006	0.5	489	978
133	1006	0.1217	119	978
166	1006	0.4785	468	978
95	1006	0.0532	52	978
98	1006	0.0624	61	978
98	1006	0.0624	61	978

202	1006	0.1636	160	978
206	1006	0.1268	124	978
535	1006	0.4734	463	978
48	1006	0.1012	99	978
236	1006	0.2055	201	978
336	1006	0.3722	364	978
335	1006	0.3773	369	978
429	1006	0.3538	346	978
.	.	.	.	.
386	1006	0.6892	674	978
.	.	.	.	.
72	1006	0.0368	36	978
316	1006	0.3354	328	978
43	1006	0.0787	77	978
